# Histopathologic brain age estimation via multiple instance learning

**DOI:** 10.1007/s00401-023-02636-3

**Published:** 2023-10-10

**Authors:** Gabriel A. Marx, Justin Kauffman, Andrew T. McKenzie, Daniel G. Koenigsberg, Cory T. McMillan, Susan Morgello, Esma Karlovich, Ricardo Insausti, Timothy E. Richardson, Jamie M. Walker, Charles L. White, Bergan M. Babrowicz, Li Shen, Ann C. McKee, Thor D. Stein, The PART Working Group, Kurt Farrell, John F. Crary

**Affiliations:** 1 Department of Pathology, Icahn School of Medicine at Mount Sinai, Friedman Brain Institute, 1 Gustave L. Levy Place, Box 1194, New York, NY 10029 USA; 2 Department of Artificial Intelligence and Human Health, Nash Family Department of Neuroscience, Ronald M. Loeb Center for Alzheimer’s Disease, Friedman Brain Institute, Neuropathology Brain Bank and Research CoRE, Icahn School of Medicine at Mount Sinai, 1 Gustave L. Levy Place, Box 1194, New York, NY 10029 USA; 3 Department of Neurology, Icahn School of Medicine at Mount Sinai, New York, NY USA; 4 Frontotemporal Degeneration Center, Perelman School of Medicine, University of Pennsylvania, Philadelphia, PA USA; 5 Nash Family Department of Neuroscience, Icahn School of Medicine at Mount Sinai, Friedman Brain Institute, New York, NY USA; 6 Human Neuroanatomy Laboratory, School of Medicine, University of Castilla‐La Mancha, Albacete, Spain; 7 Department of Pathology, University of Texas Southwestern Medical Center, Dallas, TX USA; 8 Department of Pathology, Alzheimer’s Disease and CTE Center, Boston University School of Medicine, Boston, MA USA; 9 Department of Veterans Affairs Medical Center, Bedford, MA USA; 10 VA Boston Healthcare System, Boston, MA USA

**Keywords:** Machine learning, Aging, Biological clock, Methylation, Digital pathology

## Abstract

Understanding age acceleration, the discordance between biological and chronological age, in the brain can reveal mechanistic insights into normal physiology as well as elucidate pathological determinants of age-related functional decline and identify early disease changes in the context of Alzheimer’s and other disorders. Histopathological whole slide images provide a wealth of pathologic data on the cellular level that can be leveraged to build deep learning models to assess age acceleration. Here, we used a collection of digitized human post-mortem hippocampal sections to develop a histological brain age estimation model. Our model predicted brain age within a mean absolute error of 5.45 ± 0.22 years, with attention weights corresponding to neuroanatomical regions vulnerable to age-related changes. We found that histopathologic brain age acceleration had significant associations with clinical and pathologic outcomes that were not found with epigenetic based measures. Our results indicate that histopathologic brain age is a powerful, independent metric for understanding factors that contribute to brain aging.

**Supplementary Information:**

The online version contains supplementary material available at 10.1007/s00401-023-02636-3.

## Introduction

The central nervous system is highly vulnerable to age-related changes; however, the boundaries between normal healthy aging and pathological neurodegenerative processes are not well understood [2, 40, 58]. Histopathologically, hallmark age-related changes in health and disease include the presence and accumulation of lipofuscin, neurofibrillary tangles, amyloid plaques, granulovacuolar degeneration, Hirano bodies, and cerebrovascular injuries, such as arteriolosclerosis, amyloid angiopathy, infarcts, and white matter rarefaction [2, 40, 50]. The impact of these individual changes and their synergistic effects on brain function and cognition are not fully understood, but evidence suggests that increased burden correlates clinically with functional impairment [31, 49]. Understanding the relationships between histopathologic change, aging, and neurodegeneration has the potential to establish effective strategies for deeper understanding of neurodegenerative mechanisms [43].

Most age-related changes in the brain occur on a continuum influenced by intrinsic and extrinsic factors. Determining what constitutes normal or pathological in the context of varying aging rates is complex. Understanding factors that lead to accelerated aging can elucidate mechanisms that increase risk of disease and death; in contrast, studying successful aging may lead to identification of protective factors. Estimating chronological age using biological inputs, such as imaging or epigenetic data, provides a potent tool for investigating factors affecting relative aging rates [11, 12, 29, 33, 64]. Discrepancies between estimated and actual chronological age quantify the degree of age acceleration or deceleration. Epigenetic clocks are a particularly promising method of age estimation that leverage patterns of DNA methylation, thought to reflect the accumulation of environmental and genetic damage over time, to predict chronological age [13, 56]. Brain age estimation models based on neuroimaging data have also been widely used, as they are able to predict chronological age using brain volume, white matter integrity, metabolic signal, or functional connectivity as inputs [4, 13, 36, 44, 60]. Through these approaches, researchers have found clinical, genetic, and environmental factors significantly associated with accelerated brain aging [22, 44, 60]. However, the resolutions of conventional neuroimaging approaches, such as MRI, are limited, only able to capture macroscopic changes occurring at the level of cell populations. In contrast, histopathological analysis provides an ability to assess cellular features (e.g., morphological changes) that are not conveyed by the magnetic properties of tissues captured by conventional neuroimaging. Thus, histopathological brain age estimation has the potential to provide a completely novel window into brain aging.

The large magnitude of data per slide represents a challenge in applying deep learning frameworks to histopathological datasets. An approach to circumventing this problem is multiple instance learning (MIL) in which each whole slide image (WSI) is broken down into small (256 × 256 pixel) tiles, encoded into feature vectors, and then merged via a pooling function to create a single slide-level representation [5, 32, 42, 66]. While this poses no problem for tasks that are dependent on the microscopic features provided in each tile, macroscopic topographic context is lost with this approach. Topography plays a crucial role in providing anatomic and spatial context in neuropathological processes. Whether preservation of these features might be useful for accurate age estimation is unclear.

In this study, we sought to characterize both histopathological and clinicopathological correlates of histopathologic brain age acceleration by training a context-aware attention-based deep multiple instance learning model on an age estimation task using WSI data stained with a routine structural stain, Luxol-fast blue (LFB) counterstained hematoxylin & eosin (LH&E), on hippocampal sections from an autopsy cohort of aged individuals (*n* = 689). To account for neuropathologically relevant topography, we addressed the utility of deploying a spatially resolved graph convolutional network (GCN) into our MIL pipeline [62]. We formulated WSIs as a graph-based data structure in the Euclidean space in which nodes correspond to individual tiles taken from the WSI and edges are connected between adjacent image tiles from the true spatial coordinates of the WSI. GCN’s have recently been introduced as a successful strategy in digital pathology in the field of oncology, but to our knowledge, this project is the first time they have been deployed in neuropathology [9, 37, 73, 74]. To validate our findings, we performed histopathologic brain age estimation on an independent dataset from a separate population (*n* = 251). We also compared observed associations to brain age acceleration derived from DNA methylation biologic clocks. This investigation aims to provide insight into the mechanisms of brain aging and the potential use of histopathological markers as indicators of brain age acceleration.

## Methods

### Dataset

Tissue sections were subjected to a comprehensive histopathological workup by neuropathologists and 93.9% of cases (619 of 659 with data on amyloid level) had no more than mild amyloid deposition (Thal phase ≤ 2 and CERAD NP score of either “absent” or “sparse”), increasing the likelihood of isolating age-dependent rather than disease-dependent signals. Stains were performed on 4 µm-thick formalin-fixed paraffin-embedded (FFPE) sections stained with Luxol-fast blue and counterstained with hematoxylin & eosin (LH&E), with one section per subject included in the analysis. Sections from the body of the hippocampus were targeted, but this neuroanatomical landmark was not represented in all sections, and there was some variability noted with regard to representation along the anterior–posterior axis. Sections mounted on positively charged slides were dried overnight. For each batch of slides stained, a known severe AD case was included as a positive staining control. WSI were scanned using an Aperio CS2 (Leica Biosystems, Wetzlar Germany) digital slide scanner at 20 × magnification. These cases also had a spectrum of age-related changes, including aging-related tau astrogliopathy, argyrophilic grains, incidental Lewy body pathology, and cerebrovascular disease, alongside other changes [31]. Neurofibrillary tangle density was calculated via a SegNet model architecture as detailed in Marx et al*. 2022 *[45].

To define cognitive impairment, we utilized a multi-faceted approach as outlined in McKenzie et al. [46]. Specifically, we examined clinical and research records for availability of a clinical dementia rating (CDR) score, mention of any clinical diagnosis suggestive of cognitive impairment in the clinical record, or Mini-Mental State Examination (MMSE) within 2 months of death. Cognitive impairment was defined as a CDR ≥ 0.5, or MMSE ≤ 24, or clinical notation of a cognitive disorder within 2 months of death [21, 51]. Brain donors with information available on any of these measures, but scoring better than the impairment threshold were included in the non-impaired group. Associated sample sizes for clinical metrics can be found in Supplementary Table 2.

### Genetics

High-throughput isolation of DNA was performed using the MagMAX DNA Multi-Sample Ultra 2.0 Kit on a KingFisher Flex robotic DNA isolation system (Thermofisher, Waltham, MA) according to the manufacturer's protocol. Briefly, 20–40 mg of fresh-frozen brain tissue was placed into a deep-well plate and treated with 480 μL of Proteinase K mix (Proteinase K, Phosphate Buffered Saline [pH 7.4], Binding Enhancer) and incubated overnight at 65 °C at 800 rpm on a shaking plate. Genomic DNA was isolated and purified using magnetic particles. DNA quality control was performed using a nanodrop spectrophotometer (concentration > 50 ng/μL, 260/280 ratio 1.7–2.2). Genotyping was performed using single-nucleotide polymorphism (SNP) microarrays (Infinium Global Screening Array v2.4. or the Infinium OmniExpress-24, Illumina, San Diego CA). Raw genotype files were converted to PLINK-compatible files using GenomeStudio software (Illumina, San Diego CA). *MAPT* haplotype was determined using the rs8070723 H2 tagging SNP and *APOE* genotype was determined using the rs429358 rs7412 tagging SNPs. For analyses, the *APOE* status was collapsed into a binary variable of the presence or absence of *APOE* ε4.

### DNA methylation

DNA was isolated from fresh-frozen frontal cortices (from paired histological sections) using an automated kingfisher DNA extractor and sent to the Children’s Hospital of Philadelphia (CHOP) Center for Applied Genomics (CAG). DNA then underwent bisulfite conversion and was interrogated using the Infinium Human MethylationEPIC BeadChip (V.1) array which measures DNA methylation at approximately 850,000 CpG sites. Samples were randomized across plates, run in duplicate, and quality controlled. Raw DNA methylation data were processed using the open-source OpenSesame pipeline [76]. Resulting *M*-Values were then used to calculate “clocks” using the MethylClock tool or using published CpG sites and weights for six DNA methylation clocks: Horvath, Hannum, PhenoAge, GrimAge, Cortical, and Zhang [26, 29, 41, 64, 72]. These clocks were selected to provide a representative range of DNA methylation age estimates using first-generation clocks trained on chronological age (e.g., Horvath, Hannum), second-generation clocks trained on age-based biomarker profiles (e.g., PhenoAge, GrimAge), and hypothesized brain-related clocks (e.g., Cortical).

### Replication dataset

Independent replication dataset was acquired utilizing the autopsy cohort of the Manhattan HIV Brain Bank. Scanned digital images of formalin-fixed paraffin-embedded (FFPE) tissue sections from the hippocampus were derived from autopsy brains from a subset of individuals from a previously described collection [52]. Images were acquired for analysis using an Olympus VS110 virtual slide scanning system and VS-ASW software (Olympus America Inc., Center Valley, PA) at 10× magnification.

### Data preprocessing

**Fig. 1:**
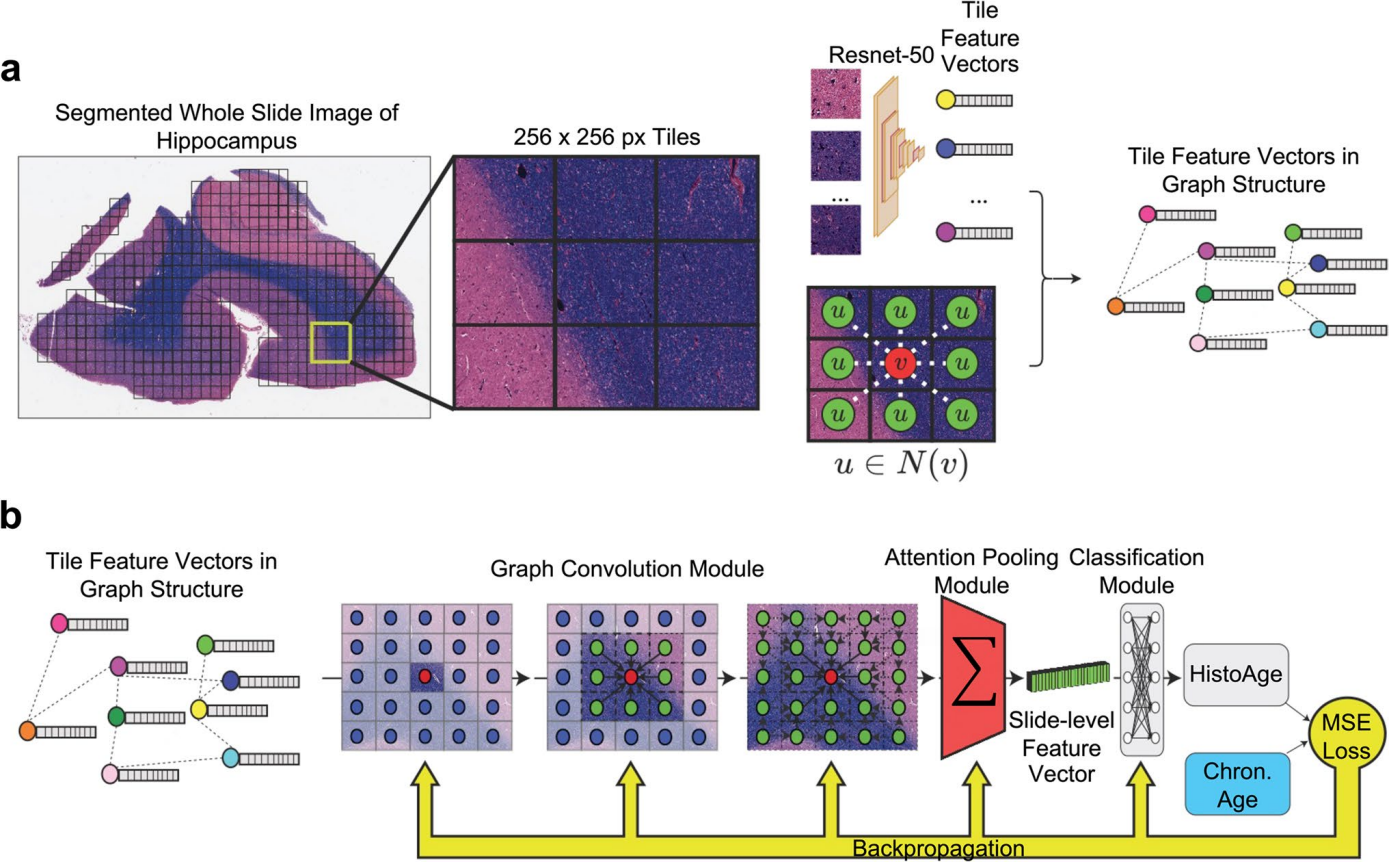
Schematic of preprocessing and model training. **a** Preprocessing: whole slide images (WSI) are segmented into 256 × 256 pixel tiles, removing areas without tissue. The tiles are then passed through an Imagenet pretrained Resnet-50 convolutional neural network, which yields feature vectors. Finally, the WSI is represented as a graph where each node is a tile encoded into a feature vector, with edges between tiles that are adjacent in slide space. **b** Model training: the WSI represented as a graph structure is passed through a graph convolution model, which learns not only the features of each tile but also messages passing between tiles, integrating macroscale information along with microscopic information. This is then passed through an attention pooling module, which pools all the tile feature vectors into a single slide-level feature vector. This is then passed through a classification model, which estimates HistoAge. HistoAge is taken with chronologic age and the mean-squared error (MSE) loss is used to update the weights of the model

Representative schematic of preprocessing workflow can be found in Fig. 1A. For a given sample, let WSI, *S*, and associated chronologic age, *Y*, be a single observation in a dataset $\{{S}_{i}, {Y}_{i}{\}^{N}}_{i=1}$. For graph construction, we used the same framework as Chen et al. [9]. To construct graph, $G$, for $S$, automatic tissue segmentation is performed following the CLAM protocol including transforming a low-downsampled version of $S$ into hue saturation value (HSV) colorspace and then using Otsu’s Binarization on the saturation channel to separate LH&E tissue from background [42, 71]. Then, non-overlapping 256 × 256 tile-level image regions at 20 × magnification are sampled and used as input for a truncated Resnet50 model pretrained on ImageNet, which extracts feature vector, $h \epsilon {\mathbb{R}}^{1x1024}$, via spatial average pooling after the third residual block [16, 28]. This is a common feature space used in many digital pathology deep learning tasks [9, 10, 42]. Thus, ${S}_{i}$ is represented as node feature matrix ${X}_{i} = \{{h}_{1};\, {h}_{2}; ..;\, {h}_{{M}_{i}}\} \epsilon {\mathbb{R}}^{{M}_{i}\times 1024}$ for ${M}_{i}$ total tiles in ${S}_{i}$. We denote the adjacency matrix of $G$ as $A = [{A}_{ij}]$ where ${A}_{ij}=1$ if the true pixel coordinates of tiles $i$ and $j$ are adjacent and ${A}_{ij}=0$ otherwise. An image patch must be connected to other patches and can be surrounded by at most 8 adjacent patches, so the sum of each row or column of A is at least one and at most 8.

### Model architecture

Schematic of model architecture can be found in Fig. 1B. First, $G$ is passed through the graph convolutional module which entails two layers of GraphSAGE convolutions each with ReLU activation functions converting $G\to G^{\prime} \epsilon {\mathbb{R}}^{{M}_{i}xd}$ where $d$ is the hidden layer dimension at 256. $G^{\prime}$ is then passed through an attention pooling module using the modified set transformer as detailed in Baek et al. 2021 with two attention heads [3, 8]. GraphSAGE convolutions were used for the layers within the modified set transformer. The attention pooling module converts $G^{\prime}\to Z \epsilon {\mathbb{R}}^{1\times d}$. $Z$ is then used as input for a fully connected classification layer to predict age ($Y^{\prime}$). The mean square error loss is based on $Y$, the chronologic age, and $Y^{\prime}$, the predicted age.

### Model training and implementation

Fifty cross-fold validation was implemented with 9:1 train-validation split ratios. For each fold, the model was trained for 100 epochs. Training was performed on an NVIDIA A-100 40 GB GPU, optimized via Adam optimization with a learning rate of 0.0005, weight decay of 0.01, dropout of 0.30, and batch size of 8 [25]. Model training and evaluation were accomplished using PyTorch deep learning library, while all graph convolutions were accomplished via PyTorch Geometric [20, 54]. Ablation experiments were performed by removing graph convolution modules and attention modules with multilayer perceptron (MLP) layers and averaging, respectively [53].

### Statistics

All statistics were carried out via the statsmodels library in Python [63]. Data were visualized using the ggplot2 package in project R [70]. Due to the known bias of age estimation models to the median age of the training set, to calculate age acceleration, age estimation values were regressed on chronologic age [39, 65]. All reported odds ratios and beta values were calculated via logistic regression and ordinary least squares, respectively, with sex and chronologic age as nuisance covariances.

## Results

### Human aging brain whole slide image dataset

A total of 689 cases were included (Table 1). The overall mean age at death was 85 years (range of 51–108) and 370 (54%) were female. 335 (49%) had cognitive impairment. Of the 362 cases with genetic data, 14% were carriers of the ε4 allele, 23% were carriers of the *APOE* ε2 allele, and 98% were carriers of the *MAPT* H1 haplotype. Neuropathologic review revealed moderate amounts of phosphorylated-tau (p-tau) burden, with a mean neurofibrillary tangle (NFT) density of 5.5 per mm^2^ and 75% having a Braak stage of II or higher. These findings support previous observations regarding the increased frequency of association between *MAPT* H1 and *APOE* ε2 with primary age-related tauopathy (PART), as well as the relative decreased frequency of *APOE* ε4 in PART cohorts [1, 15, 47, 59, 61, 68]. Clinical and neuropathological inclusion and exclusion criteria have been reported previously [19].

**Table 1:** Dataset metrics and association with HistoAge acceleration

	Cases (*n*)	Mean (SD) or % (*n*)	Association with HistoAge acceleration	*p* value
Demographic				
Female sex	689	54% (370)	*T* = 2.04	0.04
Age at death	689	85 (10.3)	*r* = 0.00	0.99
Years of education	66	15.0 (2.8)	*r* = −0.02	0.86
Clinical				
Cognitive impairment	689	49% (335)	*T* = 3.98	0.002
CDR	297		ρ = 0.13	0.04
0		44% (130)		
0.5		21% (63)		
1		13% (38)		
2		10% (29)		
3		13% (37)		
MMSE	240	25.6 (4.5)	*r* = −0.11	0.02
Diagnosis of impairment	538	47% (251)	*T* = 3.35	> 0.001
Genetics				
*APOE* genotype	362		*F* = 1.56	0.19
ε2/ε2		1% (4)		
ε2/ε3		22% (78)		
ε2/ε4		0% (0)		
ε3/ε3		63% (229)		
ε3/ε4		13% (46)		
ε4/ε4		1% (5)		
ε2 carrier		23% (82)	*T* = 0.17	0.87
ε4 carrier		14% (51)	*T* = −0.12	0.90
*MAPT* genotype	362		*F* = 0.78	0.46
H1/H1		65% (235)		
H1/H2		33% (118)		
H2/H2		3% (9)		
H1 carrier		98% (353)	*T* = −0.56	0.58
H2 carrier		35% (127)	*T* = 1.22	0.23
Epigenetics				
DNA methylation clocks	293			
Horvath clock age		74.6 (10.5)	*r* = 0.04	0.49
Hannum clock age		60.0 (9.8)	*r* = 0.00	0.99
PhenoAge clock age		6.0 (7.9)	*r* = −0.03	0.59
GrimAge clock age		91.1 (7.6)	*r* = 0.01	0.94
Cortical clock age		90.2 (9.7)	*r* = 0.09	0.14
Zhang clock age		57.7 (5.0)	*r* = 0.00	0.99
Neuropathology				
Braak stage	689		ρ = 0.13	** > 0.001**
0		9% (61)		
I		16% (111)		
II		27% (183)		
III		26% (180)		
IV		18% (124)		
V		4% (30)		
NFT density (n/mm^2^)	677	5.5 (6.1)	*r* = 0.14	** > 0.001**
Argyrophilic grain disease	210	16% (33)	*T* = 3.26	**0.001**
Aging-related tau astrogliopathy	603	27% (160)	*T* = 3.46	** > 0.001**
Lewy bodies	552	10% (54)	*T* = −2.05	**0.04**
TDP-43	170	5% (8)	*T* = −0.22	0.83
Neuritic plaques	644	6% (40)	*T* = −1.25	0.21
Diffuse plaques	652	41% (266)	*T* = 0.47	0.64
Cerebrovascular disease	227	18% (41)	*T* = 2.35	**0.02**
Cerebral amyloid angiopathy	513	20% (101)	*T* = 1.23	0.22

Associations between variables are assessed via two-sample *t *test (*T*), one-way ANOVA (*F*), Pearson correlation (*r*), or Spearman correlation (ρ). Bold values indicate *p* < 0.05

Argyrophilic grain disease was present in 16% (33 out of 210) of the cases assessed. 27% (160 out of 603) of the cases showed evidence of aging-related tau astrogliopathy, and 10% (54 out of 552) had Lewy body inclusions. TDP-43 inclusions were found in 5% (8 out of 170) of the cases assessed. This dataset had mild amyloid burden with the vast majority of the cases qualifying as either “definite” or “possible” PART [15, 69], with 6% (40 out of 644) of the cases exhibiting any neuritic plaques and 41% (266 out of 652) showing any diffuse amyloid plaques. Only 3.3% (21 out of 644) of the cases exhibited neuritic plaques with a CERAD NP score of “moderate” or “frequent” and 4.6% (30 out of 659) showed diffuse amyloid plaques > Thal phase 2. 18% (41 out of 227) of the cases assessed had evidence of cerebrovascular disease and 20% (101 out of 513) showed signs of cerebral amyloid angiopathy. To avoid biasing the model, cases which met neuropathologic diagnostic criteria of more than mild Alzheimer’s disease pathology were excluded, even though this is not entirely representative of the population [35].

### Context-aware attention-based multiple instance learning model training estimation of histopathologic brain age

Our histopathologic brain age estimation (HistoAge) model was trained on 689 uniformly processed digitized whole slide images (WSI) of post-mortem human hippocampal sections from aged individuals stained with LH&E using a multiple instance learning (MIL) framework [31, 45, 46, 69]. The hippocampus is known to be involved in both brain aging and age-dependent neurodegenerative disease and thus is an ideal anatomic region for this analysis [40].

We deployed a novel MIL model architecture (Fig. 1) utilizing graph convolutional networks (GCN) representing each slide as a Euclidean graph. The WSI is segmented into tile images (256 × 256 pixels) which represents the nodes of the network and each tile is connected to its adjacent neighbor in slide space. Constructing graphs in Euclidean space enables us to leverage spatial convolutions that perform a spatial aggregation function similar to convolutional neural networks (CNN), to encode WSI topographic information into the model. Neighboring tiles were detected by representing the top-left pixel coordinates of each tile in a 2D-point cloud fed into a kd-tree and queried all points within a radius of 256 * √2 which is the maximum distance between two adjacent tiles (the diagonal distance of one tile).

To pool our network of tile-level feature vectors into a single WSI-level feature vector, we used MIL attention, a technique wherein the model learns an optimal weighted summation of the tiles to represent the slides [32]. In this framework, tiles with a higher attention value were learned by the model to best represent the slide. We thus inferred that the model found these areas of the slide to be most relevant to histopathologic brain age estimation and thus likely represented relevant features of age-dependent histology. We used a modified set transformer for our specific attention algorithm [3].

**Fig. 2:**
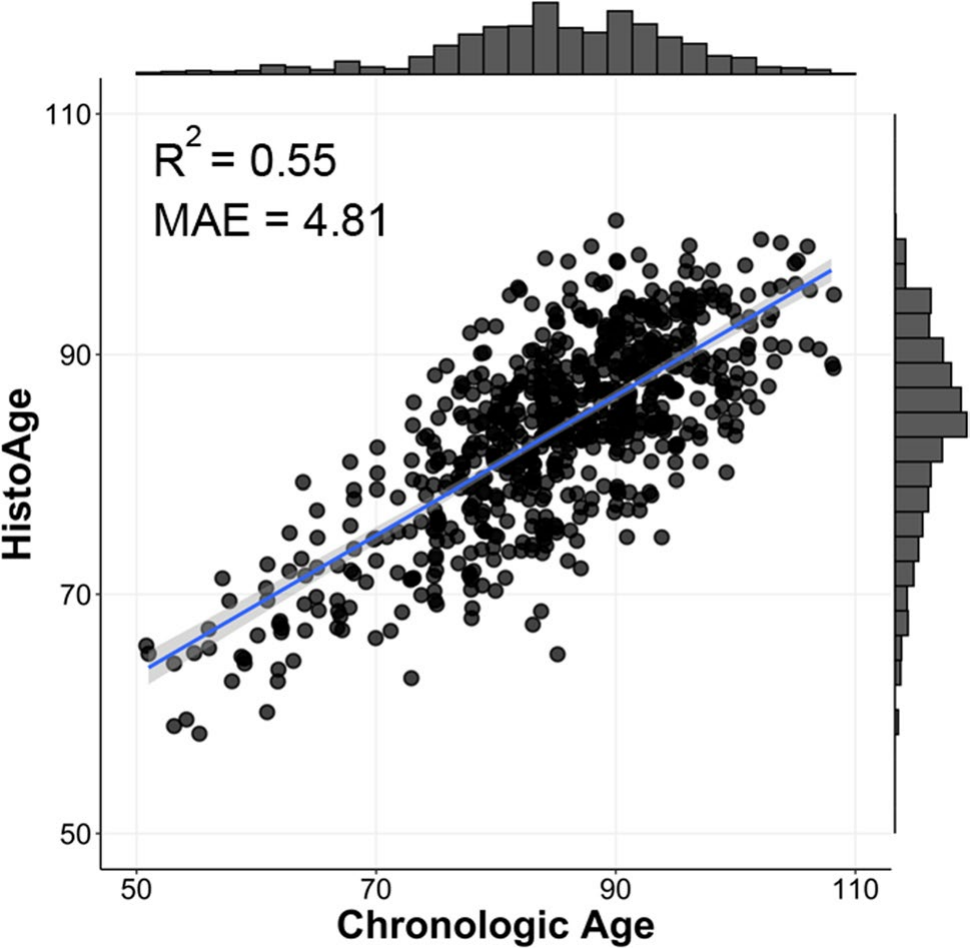
HistoAge is highly correlated with chronological age. Estimated HistoAge plotted against chronologic age with corresponding marginal histograms. The blue line represents the best-fit linear regression line between HistoAge and chronologic age, and the residuals from this line are HistoAge acceleration. The coefficient of determination and mean absolute error (MAE) are shown

Our model accurately estimated the age at death with a mean absolute error of 5.45 ± 0.22 across 50 cross-fold validations. This performance was superior to the results obtained from ablated models, which involved the removal of graph convolution and attention modules (Table 2). All subsequent model results and inferences are from the single best-performing model fold which had a mean absolute error of 4.81 (Fig. 2). The Pearson correlation between HistoAge and chronologic age was 0.74 (*p* < 0.001). Altogether, these results indicate that context-aware attention-based multiple instance learning can successfully estimate histopathologic brain age.

**Table 2:** Model performance and ablation analysis

Graph	Attention	MAE	RMSE	MSE
−	−	6.87 ± 0.18	8.71 ± 0.24	75.91 ± 4.47
−	+	5.84 ± 0.17	7.30 ± 0.22	53.33 ± 3.26
+	−	6.51 ± 0.08	8.22 ± 0.11	67.61 ± 1.87
+	+	**5.45 ± 0.22**	**6.80 ± 0.27**	**46.37 ± 3.65**

50 cross-fold runs for the final model and ablation analyses. The final model used in bold

Mean and standard deviations are shown

*MAE* mean absolute error; *RMSE* root-mean-square error; *MSE* mean-squared error

### HistoAge model attention weights are preferentially distributed in age-vulnerable neuroanatomical regions

**Fig. 3:**
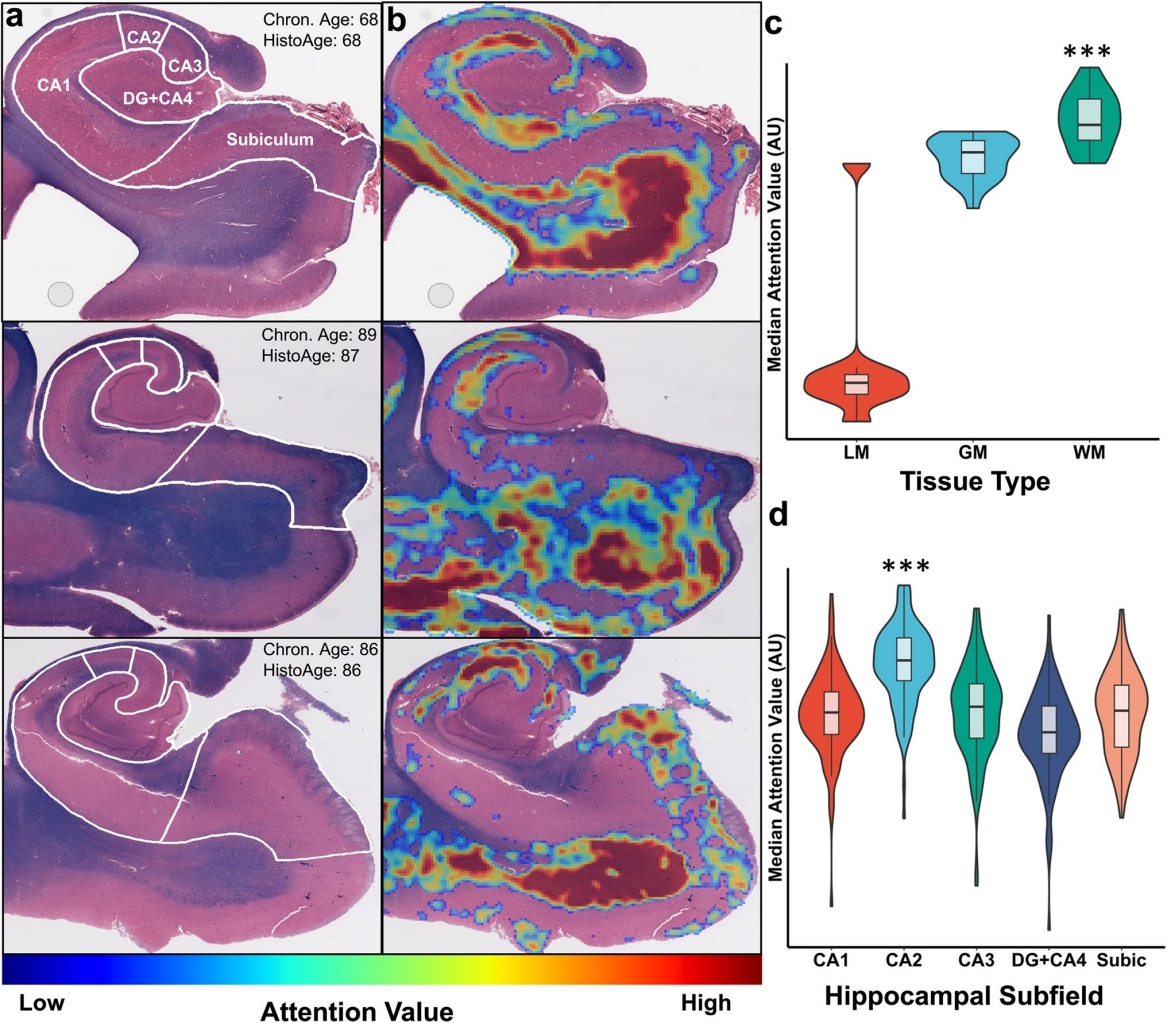
HistoAge model exhibits increased attention in vulnerable neuroanatomical regions. **a** Three representative Luxol-fast blue counterstained hematoxylin & eosin (H&E)-stained hippocampus sections with subfield labels, chronologic age, and estimated HistoAge are displayed. **b** Corresponding attention-weighted image, where high attention values are shown in red and low attention values are shown in blue. **c** Distribution of attention levels by tissue type. WM had (*p* < 0.001) higher attention than both GM and LM and GM had significantly (*p* < 0.001) higher attention than LM. **d** Distribution of median attention values by hippocampal subfield, with the median attention value for each subfield displayed. One-way ANOVA of the distributions had an *F* = 78.9, *p* < 0.001. Subsequent Tukey’s test revealed CA2 to have significantly (*p* < 0.001) higher attention than all other subfields. *DG* dentate gyrus; *Subic* subiculum; *LM* leptomeninges; *GM* gray matter; *WM* white matter

Our model learned-attention values for each tile to identify the most salient features in estimating the age at death. Tiles with low or no attention values have little impact on the model’s computations, while tiles with high attention values are given more weight. We used a multi-headed attention module with two attention heads, because model performance was found to be superior to one or more than two attention heads (Supplemental Table 1, online resource), each head produced highly correlated attention values with near identical associations (Supplemental Fig. 1, online resource). Therefore, all attention analyses and figures used the mean attention value per tile between the two heads. Representative attention heatmaps, which visualize the attention values at each tile (Fig. 3b), wherein high levels of attention are shown in red and low are shown in blue. To gain insights into the distribution of attention values, a random selection of 21 cases were selected for hand-drawn tissue segmentation, annotating regions of gray matter, white matter, and leptomeninges (Supplemental Fig. 4, online resource). Analysis of median attention values per tissue type showed significantly (*p* < 0.001) higher attention values in white matter compared to gray matter and leptomeninges and significantly (*p* < 0.001) higher attention values in gray matter compared to leptomeninges (Fig. 3c). In addition, we analyzed the data using expert annotations of hippocampal subfields on a randomly selected subset of 111 cases [34]. We obtained the median attention value per subfield per subject, taking into consideration the variability of area in each subfield (Fig. 3d). The highest median attention of all subfields was in CA2 (one-way ANOVA, *F* = 78.9, *p* < 0.001, Tukey test *p* < 0.001). The spatial overlap between our model’s attention values and neuroanatomic regions known to be vulnerable to age-dependent change strongly suggest that our model’s approach to age estimation is using age relevant biological signals rather than over-fitting the dataset based on non-relevant features.

### Epigenetic age acceleration is not correlated with HistoAge acceleration

**Fig. 4:**
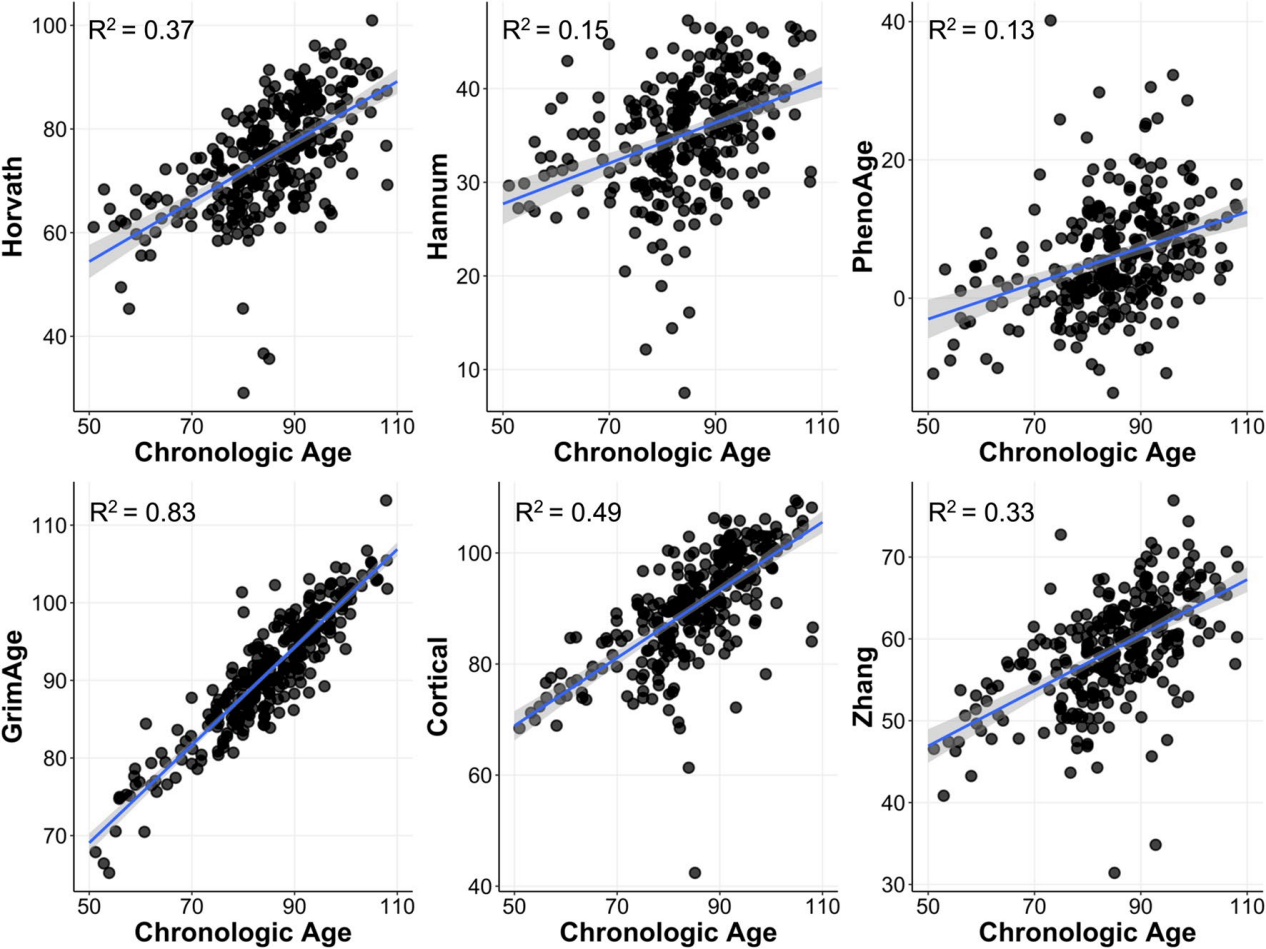
DNA methylation clocks and chronologic age. Relationships between DNA methylation clocks and chronologic age are shown. Linear regression shown (blue) and coefficients of determinations are shown in the corner (*n* = 293). Chronological age represents age of death (yr)

DNA methylation is a benchmark that is widely deployed for age estimation [29, 64]. As a next step, we wanted to evaluate how HistoAge acceleration performed in comparison to DNA methylation-based approaches using DNA isolated from the frontal cortex of matched subjects. We also wanted to understand the extent to which histopathologic aging is captured by age-dependent epigenetic changes. The correlations between chronologic age and DNA methylation clocks in a subsample of 293 cases are presented in Fig. 4. Results indicate strong correlations (*p* < 0.001) between chronologic age and Horvath (*r* = 0.61), Hannum (*r* = 0.39), PhenoAge (*r* = 0.36), GrimAge (*r* = 0.91), Cortical (*r* = 0.70), and Zhang (*r* = 0.57) DNA methylation clocks. There were strong correlations (*p* < 0.001) between HistoAge and Horvath (*r* = 0.47), Hannum (*r* = 0.29), PhenoAge (*r* = 0.25), GrimAge (*r* = 0.67), Cortical (*r* = 0.57), and Zhang (*r* = 0.52). These findings are expected given the design of these models.

Next, we examined age acceleration. Supplementary Fig. 2 (online resource) shows the relationships between all the age acceleration metrics. HistoAge acceleration was not significantly correlated with any of the DNA methylation-based age acceleration metrics. Horvath acceleration was significantly correlated with Hannum acceleration (*r* = 0.59), PhenoAge acceleration (*r* = 0.21), Cortical (*r* = 0.63), and Zhang (*r* = 0.56). Hannum acceleration was significantly correlated with PhenoAge acceleration (*r* = 0.17), GrimAge acceleration (*r* = 0.13), Cortical acceleration (*r* = 0.70), and Zhang acceleration (*r* = 0.60). PhenoAge acceleration was significantly correlated with Cortical acceleration (*r* = 0.22) and Zhang acceleration (*r* = 0.49). Cortical acceleration was significantly correlated with Zhang acceleration (*r* = 0.67). Thus, we can infer that our histopathologic brain age model captures unique features of age-dependent change not captured by DNA methylation-based approaches.

### HistoAge acceleration is significantly associated with measures of cognitive impairment

Table 1 presents the associations between HistoAge acceleration and demographic, clinical, and genetic markers. Results reveal that HistoAge acceleration was significantly higher in females compared to males (*p* = 0.04). On the other hand, GrimAge acceleration was found to be significantly higher in males compared to females (*p* < 0.001). No significant differences were observed in the other DNA methylation clock accelerations between the sexes. No significant correlations were found between any of the age acceleration markers and the *APOE* allelic and *MAPT* haplotype statuses.

**Fig. 5:**
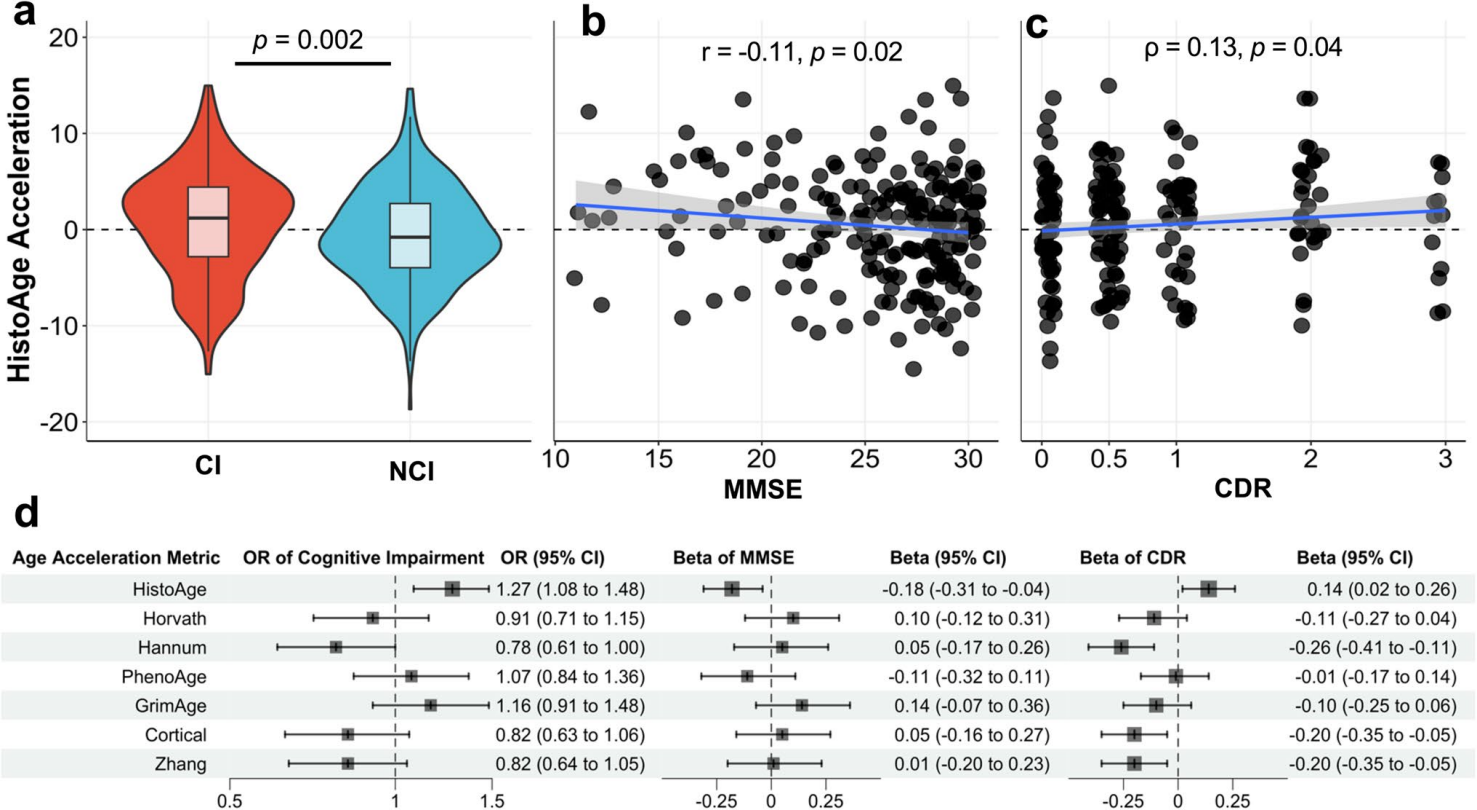
Clinicopathologic correlations with HistoAge and DNAm acceleration. HistoAge acceleration was significantly associated with antemortem cognitive impairment. **a** A loose composite label of cognitive impairment showed significantly increased HistoAge acceleration in the cognitively impaired (CI) group compared to the non-cognitively impaired (NCI) group (*p* = 0.002). **b** Pearson correlation analysis showed a modest yet significant negative correlation between HistoAge acceleration and MMSE (*p* = 0.02, *r* = −0.11). **c** Spearman correlation analysis showed a modest yet significant positive correlation between HistoAge acceleration and CDR (*p* = 0.04, rho = 0.13). **d** Modeled associations between antemortem cognitive impairment and all measures of age acceleration (HistoAge and DNA methylation-based clocks) with chronologic age and sex as nuisance covariates. *CDR* Clinical Dementia Rating; *MMSE*, Mini-Mental State Examination. Sample sizes for each comparison can be found in Supplementary Table 2 (online resource)

Additionally, there were significant relationships between all measures of cognitive impairment and HistoAge acceleration (Fig. 5). The only significant correlation between the measures of cognitive impairment and DNA methylation clock acceleration was found between Hannum and CDR (β  = −0.26, *p* < 0.001), suggesting that increased Hannum age acceleration was associated with decreased impairment. Associated sample size for clinical metrics with DNA methylation data can be found in Supplementary Table 2 (online resource). Taken together, these findings indicate that HistoAge acceleration can be used as a metric in clinicopathologic correlation analysis.

### HistoAge acceleration is significantly associated with the presence of age-related neuropathologic features

Table 1 highlights the significant associations between HistoAge acceleration and various neuropathologic variables. The results demonstrate that HistoAge acceleration was found to be significantly (*p* < 0.001) associated with several p-tau-based metrics, including Braak stage, medial temporal lobe NFT burden, argyrophilic grain disease, and aging-related tau astrogliopathy. Additionally, the presence of cerebrovascular disease was found to be significantly associated with increased HistoAge acceleration (*p* = 0.02). Cases with Lewy body inclusions showed decreased HistoAge acceleration; however, this association lost significance after controlling for age and sex (OR = 0.76, CI 0.57–1.01).

Modeled associations between all age acceleration metrics and neuropathologic variables are shown in Table 3. Both Horvath age acceleration (β = 0.15, CI 0.02–0.28) and Cortical age acceleration (β = 0.15, CI 0.02–0.28) were significantly associated with Braak Stage. Hannum age acceleration was linked to cerebrovascular disease (OR 2.83, CI 1.02–7.84) and Lewy body inclusions (OR 1.68, CI 1.05–2.70). PhenoAge acceleration was associated with TDP-43 inclusions (OR 10.27, CI 1.13–93.11), and Zhang age acceleration showed a significant association with Lewy body inclusions (OR 2.05, CI 1.26–3.34). Associated *n*’s for neuropathologic variables with DNA methylation data can be found in Supplementary Table 2 (online resource). These results highlight the potential of DNA methylation clocks as valuable markers of enhanced risk for neurodegeneration.

**Table 3:** Association between age acceleration and neuropathologic variables

	HistoAge acceleration		DNA methylation clock age acceleration					
			Horvath	Hannum	PhenoAge	GrimAge	Cortical	Zhang
Vascular pathology								
Cerebrovascular disease	**1.67 (1.13–2.47)**		1.38 (0.73–2.61)	**2.83 (1.02–7.84)**	0.67 (0.35–1.29)	1.27 (0.64–2.54)	2.14 (0.92–4.98)	1.76 (0.95–3.23)
Cerebral amyloid angiopathy	1.17 (0.93–1.46)		1.43 (0.93–2.19)	1.45 (0.95–2.21)	1.06 (0.73–1.53)	0.89 (0.61–1.31)	1.32 (0.85–2.06)	1.44 (0.95–2.19)
pTau Pathology								
Braak stage*	**0.14 (0.06–0.22)**		**0.15 (0.02–0.28)**	0.09 (−0.04–0.22)	−0.03 (−0.16–0.10)	−0.04 (-0.16–0.09)	**0.13 (0.00–0.26)**	0.04 (−0.09–0.17)
Neurofibrillary tangle density*	**0.82 (0.39–1.24)**		0.23 (−0.35–0.81)	−0.11 (−0.70–0.47)	−0.39 (−0.97–0.20)	−0.24 (−0.83–0.35)	0.18 (−0.40–0.77)	−0.10 (−0.69–0.48)
ARTAG	**1.48 (1.22–1.80)**		0.95 (0.73–1.22)	0.84 (0.64–1.09)	1.04 (0.80–1.35)	1.08 (0.83–1.41)	0.83 (0.64–1.07)	0.87 (0.67–1.12)
Argyrophilic grain disease	**2.01 (1.32–3.06)**		1.53 (0.28–8.36)	1.22 (0.27–5.46)	1.89 (0.41–8.75)	1.69 (0.40–7.12)	7.06 (0.37–136)	1.93 (0.33–11.13)
Other proteinopathy								
Lewy bodies	0.76 (0.57–1.01)		1.05 (0.69–1.60)	1.68 (1.05–2.70)	1.31 (0.88–1.94)	1.38 (0.92–2.07)	1.51 (0.90–2.53)	2.05 (1.26–3.34)
TDP-43 positive	1.09 (0.53–2.25)		0.73 (0.26–2.03)	1.24 (0.31–4.91)	**10.2 (1.10–93.1)**	0.19 (0.03–1.13)	4.15 (0.70–24.4)	2.60 (0.40–24.0)
Diffuse amyloid plaques	1.25 (0.95–1.74)		0.90 (0.30–2.68)	0.80 (0.29–2.18)	0.62 (0.17–2.34)	1.87 (0.73–4.79)	1.02 (0.32–3.28)	0.90 (0.29–2.76)
Neuritic plaques	0.96 (0.82–1.12)		1.20 (0.93–1.55)	0.99 (0.78–1.26)	0.87 (0.68–1.11)	1.20 (0.94–1.55)	0.92 (0.72–1.17)	1.05 (0.83–1.34)

Modeled relationships between neuropathologic variables and age acceleration variables via logistic regression. Sex and chronologic age were used as nuisance covariates. Odds ratios (95% CI)

Bold values indicate *p* < 0.05

*Continuous values, OLS model with β values (95% CI)

### HistoAge model predicts age in an independent replication cohort

The efficacy of the HistoAge paradigm was evaluated by training a new model on an independent dataset of 251 individuals. Demographic, genetic, and neuropathologic variables for this replication cohort are summarized in Table 4. The model yielded a mean absolute error of 7.81 ± 0.53, root mean-squared error of 10.1 ± 0.54, and mean-squared error of 102.3 ± 11.3. The best-performing cross-validation fold demonstrated a mean absolute error of 7.22 and a strong Pearson correlation (*r* = 0.61, *p* < 0.001) between HistoAge and chronological age (Supplemental Fig. 3 online resource). Results indicate significant associations between increased HistoAge acceleration and the presence of argyrophilic grain disease (*T* = 4.03, *p* < 0.001), amyloid neuritic plaques (*T* = 1.98, *p* = 0.049), amyloid diffuse plaques (*T* = 2.40, *p* = 0.017), and cerebrovascular disease (*T* = 1.99, *p* = 0.048). These significant neuropathologic feature associations with HistoAge acceleration in a dataset with a smaller *n*, younger mean age at death and larger age range, lower resolution strongly suggests that this approach can be expanded to smaller cohorts for future analyses on disease populations with less accessible data.

**Table 4:** Replication dataset demographic, genetic, and neuropathologic variables and HistoAge acceleration

	Mean (SD) or % (*n*)	Association with HistoAge acceleration	*p*
Demographic			
Female sex	38% (96)	*T* = −1.33	0.186
Age at death	53.1 (12)	*r* = 0.00	0.999
Genetics			
APOE genotype		*F* = 0.45	0.814
ε2/ε2	0.4% (1)		
ε2/ε3	23% (34)		
ε2/ε4	4% (11)		
ε3/ε3	56% (140)		
ε3/ε4	23% (58)		
ε4/ε4	2% (6)		
ε2 carrier	18% (46)	*T* = −0.32	0.748
ε4 carrier	30% (75)	*T* = 0.18	0.855
Neuropathology			
Braak stage (collapsed)		ρ = 0.07	0.294
0	37% (92)		
I or II	18% (44)		
III or IV	15% (37)		
Neurofibrillary tangles	24% (59)	*T* = 1.26	0.210
Argyrophilic grain disease	7% (17)	*T* = 4.03	** > 0.001**
Aging-related tau astrogliopathy	5% (13)	*T* = 1.67	0.096
Neuritic plaques	10% (24)	*T* = 1.98	**0.049**
Diffuse plaques	33% (82)	*T* = 2.40	**0.017**
Cerebrovascular disease	18% (46)	*T* = 1.99	**0.048**
Cerebral amyloid angiopathy	6% (16)	*T* = 1.03	0.305

Associations between variables are assessed via two-sample *t* test (*T*), one-way ANOVA (*F*), Pearson correlation (*r*), or Spearman correlation (ρ)

## Discussion

The aging brain undergoes complex structural and cellular changes that can impact function and increase susceptibility to neurodegenerative diseases. Gaining a deeper understanding of these changes is essential for advancing our knowledge of the underlying mechanisms involved in brain aging and the development of age-related disorders. Histopathological analysis offers the opportunity to examine tissue structure and morphology at a microscopic level, providing valuable insights into the biological processes driving these alterations. Previous approaches to studying brain aging and neurodegenerative diseases have been limited in their ability to comprehensively quantify pathological brain aging at the histologic level. In this study, we aimed to address this gap by developing a novel deep learning approach, HistoAge, for histopathological brain age estimation. Our data suggest that HistoAge may offer a holistic quantitative metric of pathologic brain aging, which has the potential to advance our understanding of the underlying mechanisms of brain aging and the development of age-related disorders. Albeit further studies leveraging whole slide images derived from populations of both individuals that have aged successfully, and those with environmental and genetic risk factors related to accelerated aging, must be conducted to elucidate the frequency and biological interpretation of HistoAge. Nonetheless, this metric has the potential to identify risk factors for pathologic aging and protective factors that contribute to successful aging, as well as to uncover novel anatomical regions vulnerable to age-related changes and new pathologic signatures of aging.

Here, we have demonstrated that our HistoAge model is effectively utilizing recognizable histological features of aging to make its estimation, providing a quantitative metric of pathologic brain aging at the histologic level. This is highlighted by the observation of high median attention values in the hippocampal CA2 subfield, which is known to be susceptible to aging-dependent changes, although it should be noted that the model did not attend to other brain regions that have been implicated in aging, such as the subiculum and dentate gyrus, and further biological interpretation is warranted [67, 69]. Further, we found that when compared to other regions, white matter was a major focus of attention, which has shown to be integral to age-dependent cognitive impairment [46]. The model’s dependence on white matter could be related to leukoaraiosis given the associations found between HistoAge acceleration and both cerebrovascular disease and cognitive impairment in this population [31, 46]. Further investigation of this relationship is warranted. The association between HistoAge acceleration and high burdens of age-related pathology further emphasizes the model’s ability to identify cellular features linked to aging and neurodegenerative diseases. Thus, HistoAge can be used in future analyses to explore pathoanatomical associations and potentially uncover novel neuropathologic features of aging.

The strong correlation between HistoAge acceleration and cognitive impairment and neuropathological features highlights the potential of this method as a metric of atypical brain aging, which is increasingly recognized as a key aspect of understanding the pathogenesis of neurodegenerative disease [18, 30]. The DNA methylation clock-based accelerations, on the other hand, showed limited and inconsistent correlations with these markers. Specifically, while Hannum and Zhang accelerations showed significant associations with some neurodegenerative markers, HistoAge had a much broader range of significant correlations, including with measures of cognitive impairment and multiple neuropathologic variables. These results underscore the superior performance of HistoAge as a tool for quantifying neuropathologic brain age acceleration.

Furthermore, using an ablation study analysis, we demonstrated that implementation of both context-aware and learned-attention pooling modules enhanced our brain age prediction model. We hypothesize that both are essential to the hierarchical representation of the WSI in which macroscale topographic information can be adequately combined with microscopic information in the whole slide representation. We believe that future machine learning approaches in digital neuropathology that are aimed at whole slide-level inference should consider incorporating graph convolutions and attention pooling to best encode both macroscopic and microscopic information into their analysis.

The results from the replication cohort demonstrate the generalizability of the HistoAge approach, despite the smaller sample size and lower resolution of the data. This indicates that meaningful insights can be obtained from smaller datasets, which is important for many autopsy studies where data collection can be challenging. However, a discrepancy was observed between the primary dataset and the replication dataset in their associations between HistoAge acceleration and neuropathologic variables. Both datasets showed significant associations between HistoAge acceleration and cerebrovascular disease and argyrophilic grain disease. However, only the replication cohort showed significant associations between HistoAge acceleration and amyloid plaques. This discrepancy might be explained by fact that the primary dataset was curated to minimize Alzheimer disease (AD) type amyloid pathology, and so is not entirely representative of the population in that age range, leaving the primary model limited in its exposure to amyloid pathology [14, 57]. Other factors, including a higher proportion of females (54% vs. 38%) and a greater mean age at death in the primary compared to the replication dataset (85 vs. 53 yr), may also have contributed to observed differences. We hypothesize that the model is conditionally aware of salient features and is dependent on the specific features of aging in the dataset it is trained on, albeit to confirm this assertation requires running the model on several more autopsy cohorts which is a limitation of this study. Nonetheless, if our hypothesis holds true, it would demonstrate that the HistoAge framework is adaptable to the unique features of aging in a specific dataset, which can be harnessed to identify risk factors specific to a particular population.

While HistoAge was strongly correlated with all DNA methylation clocks, HistoAge acceleration was not correlated with any of the DNA methylation-based age acceleration metrics. While it is not fully understood what cellular mechanisms and exposures drive DNA methylation-based age acceleration, this result indicates that those factors may not play a role in hippocampal histopathology. This contrasts with Grodstein et al. 2021 who found significant associations between increases in Cortical clock age (a DNA methylation clock specifically designed for frontal brain tissue) and AD pathology, *APOE* ε4 carrier status, and cognitive impairment [24]. In our primary analysis, neither AD pathology nor *APOE* ε4 carrier status were associated with either HistoAge acceleration or cortical age acceleration. We believe this may be due to the low prevalence of AD pathology and *APOE* ε4 in our primary dataset, or alternatively the fact that DNA methylation age acceleration was measured using DNA from the frontal cortices, whereas HistoAge acceleration was quantified in the hippocampus. This dependence is important to consider in future analyses when using age estimator modeling to analyze features associated with age acceleration.

When comparing our histopathologic brain age estimation to radiologic approaches, we did not reach the same level of accuracy. Beheshti et al. 2019 achieved a mean absolute error of 4.86 when performing brain age estimation via MRI in a similar cohort (age range 40–90 years) [4]. However, this difference is better understood when appreciating the fundamental differences between the two modalities. One obstacle in histopathologic brain age estimation is all brain tissue being examined histopathologically is sampled from deceased patients, potentially confounding the targeted signal with factors contributing to the patient’s death. Younger individuals in the dataset are likely to have decreased age-dependent pathologic changes; however, they are also more likely to show other pathologic changes related to dying at a younger age. As a result, the signal captured in a histopathologic brain age estimator could potentially include age-independent signals that led to individuals who died at an early age. However, it can be argued that these same signals could be signatures of accelerated aging. Strong associations between HistoAge acceleration and known neuropathologic signatures of aging indicate that the model is successfully modeling accumulation of age-dependent pathology.

Furthermore, radiologic analyses can take information from the entire brain, while histopathologic analyses are only privy to a specific anatomic section. It would be safe to assume that if radiologic approaches only received information from a single hippocampus per subject, they would not be able to perform at their current level of accuracy. Not only is our current approach confined to a single anatomic region, but a single cross-sectional slice of said anatomic region at that. Lee et al. (2022) performed spatial occlusion sensitivity analyses on both MRI- and fluorodeoxyglucose (FDG)-positron emission tomography (PET)-based age estimation models to identify regions most salient to their models [36]. They found that within the older aged groups, the superior temporal pole, ventricular boundary, and cerebellomedullary cistern were most contributory for the MRI-based model and inferior frontal cortices, pons, and thalamus were most contributory for the FDG PET-based model. Future approaches to the HistoAge framework will explore the implementation of a single case being represented by multiple regions in an effort to better represent brain wide histopathology.

A major limitation of our analysis was the added variance in sliced section preparation. Nationwide Alzheimer Disease Research Centers (ADRC) have no shared standard protocol for the exact position for hippocampal sections to be harvested along the anterior–posterior axis. Hippocampal anatomy, structure, and function have a considerable amount of variance along the anterior–posterior axis, and research has found differential age-dependent vulnerability across the axis, as well [17, 38]. In addition, there was variability in which hemisphere the hippocampus was sampled from for slide mounting. Research has found asymmetric vulnerability in age-related change [55, 75]. While the literature remains conflicted on which hemisphere is more vulnerable, documented differential age-dependent change between the two hemispheres suggests that incorporating random hippocampal hemispheres adds unnecessary variance to our model. A further limitation of our study is the variance in slide quality across the dataset. Artifacts in tissue slicing and mounting result in random faults across the dataset. Such faults include missing regions, addition of unnecessary tissue from other neighboring brain regions, tears in the tissue, and slide mounting artifacts such as tissue folding, tissue shearing, air bubbles, and staining artifacts. While many of these are inherent in histopathologic data, they are presumed to be randomly distributed across the dataset and not sources of bias in model training.

Despite these limitations, age estimation via histopathology provides a great deal of additional information compared to radiologic approaches. While current approaches with radiological inference are strongly suited toward measuring differences in tissue volume and thickness, histopathological analysis provides a direct insight into tissue structure and morphology. Changes at the cellular and subcellular level are much more readily analyzed via histopathological data than the inference provided by radiology. Not only can histopathology assess the degree of volume loss, but it can in turn observe and definitively diagnose the aberrant cellular morphology that the volume loss is ultimately secondary to. Furthermore, histopathologic data provides information regarding injury, inflammatory response, and degeneration. For these reasons, developing models trained on histopathological images can provide novel insight into brain aging based on microscopic features.

Moving forward, we aim to apply the HistoAge framework to a range of pathologies and disease populations, with the goal of gaining a deeper understanding of the connection between brain aging and neurodegeneration. By exploring the neuropathologic basis of various disorders and their clinicopathologic relationships, we expect to gain novel insights that could advance our understanding of these conditions. Our findings highlight the potential of HistoAge as a valuable tool in the study of brain aging and neurodegeneration, and we look forward to exploring its utility further.

Future work will expand the current histopathologic modeling approach. We will incorporate additional feature spaces via self-supervised representation learning (BYOL, DINO, SwAV, and ViT-MAE) which have shown to be effective approaches in medical imaging frameworks, as well as feature spaces from supervised-tile-level tasks more relevant to neuropathology than ImageNet classification [6, 7, 23, 27, 48]. Furthermore, we would seek to deploy this model on other subregions of the hippocampal formation (e.g., different layers, entorhinal cortex, etc.) and other non-hippocampal regions as well as other stains. We are interested in restructuring the model to accommodate multimodal information in which a single subject can be represented by multiple slides from different stains and brain regions. However, this significantly increases the dimensionality of the dataset, and thus, this would necessitate more training data.

In conclusion, the HistoAge framework provides a novel approach for estimating brain age from histology images. The results from both the primary and replication datasets demonstrate the robustness of our model in identifying histological signatures of aging and their associations with various neuropathologic variables. This highlights the potential for HistoAge to be a valuable tool for understanding the underlying mechanisms of brain aging and the development of neurodegenerative diseases. Furthermore, the adaptability of the model to different datasets implies the potential for wide-ranging applications in the study of brain aging. Finally, we also propose that this approach can be applied to other organ systems to address additional dimensions of aging. As further research is conducted, the potential of HistoAge to provide new insights into the complex relationships between aging and associated diseases will be increasingly apparent.

### Supplementary Information

Below is the link to the electronic supplementary material.

Supplementary file1 (DOCX 2960 KB)

Supplementary file2 (DOCX 14 KB)

Supplementary file3 (DOCX 14 KB)
