# Supplementary material for: Histopathologic brain age estimation via multiple instance learning
Source: Acta Neuropathol. 2023 Oct 10;146(6):785–802. doi: 10.1007/s00401-023-02636-3 (PMC10627911; doi:10.1007/s00401-023-02636-3)
Supplement: Supplementary file 1 — Supplementary file1 (DOCX 2960 KB) [file 401_2023_2636_MOESM1_ESM.docx]

**
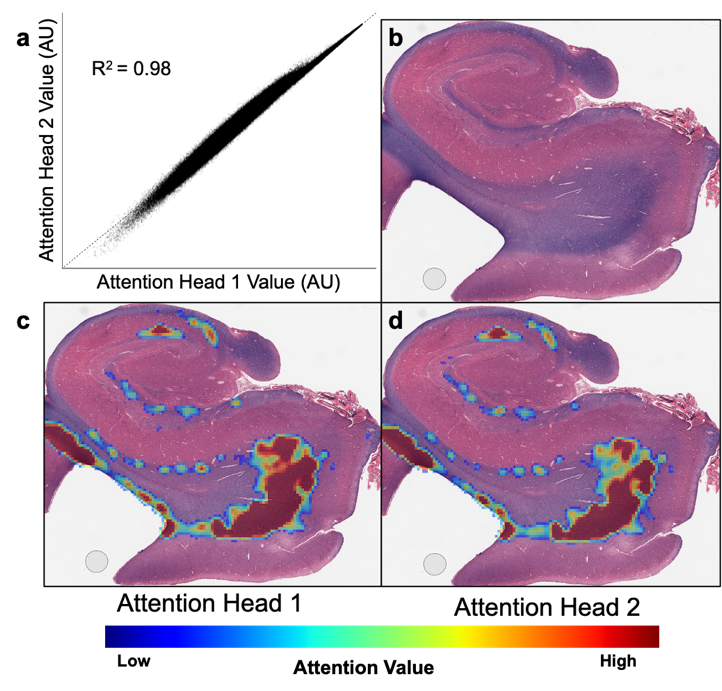
**

# Supplementary Fig. 1 | High Correlation Between Attention Heads

(**a**) Scatter plot of per tile attention values for each attention head for a random subset of 422,747 tiles. The two attention heads are highly correlated with a coefficient of determination of 0.98. Dashed line denotes the identity line. Dashed line denotes the identity line. (**b**) Representative hippocampal section with associated attention values from (**c**) attention head 1 and attention head 2 (**d**).

**
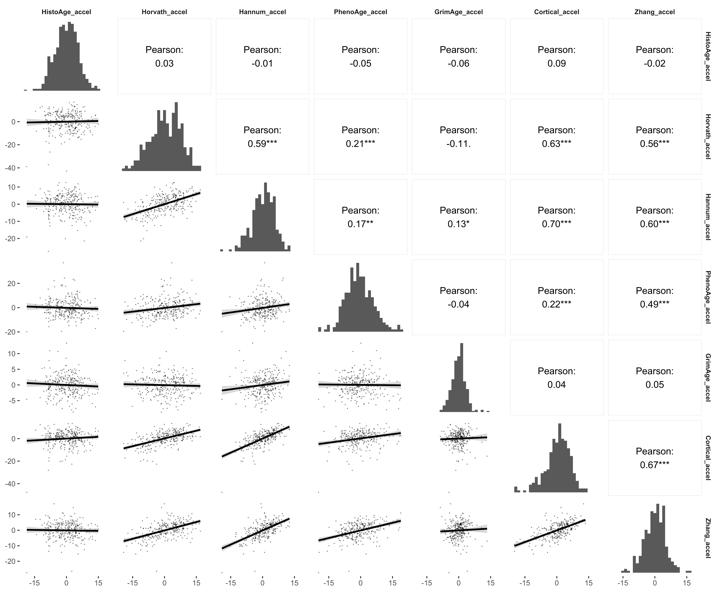
**

#

# Supplementary Fig. 2 | Correlogram of Age Acceleration Metrics

Regression plots between each age acceleration metric. Pearson correlations are shown with level of significance shown as * *p* < 0.05, ** *p* < 0.01, *** *p* < 0.001 (*n*=293)


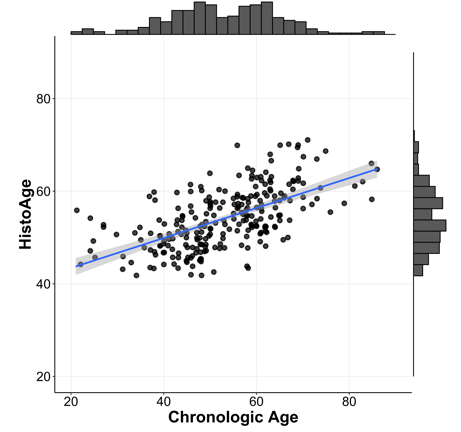


R^2^ = 0.37

MAE = 7.22


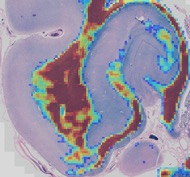

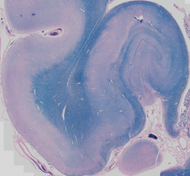

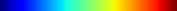


**Attention Value**

**High**

**Low**

**a**

**b**

**c**

Chron. Age: 70

HistoAge: 59

# Supplementary Fig. 3 | Independent Replication Cohort Model Performance and Attention Distribution

(**a**) Estimated HistoAge plotted against chronologic age in independent replication cohort with corresponding marginal histograms. The blue line represents the best fit linear regression. Coefficient of determination and mean absolute error (MAE) are shown. (**b**) Representative hippocampal section from the independent replication dataset with (**c**) associated attention values (*n*=251).


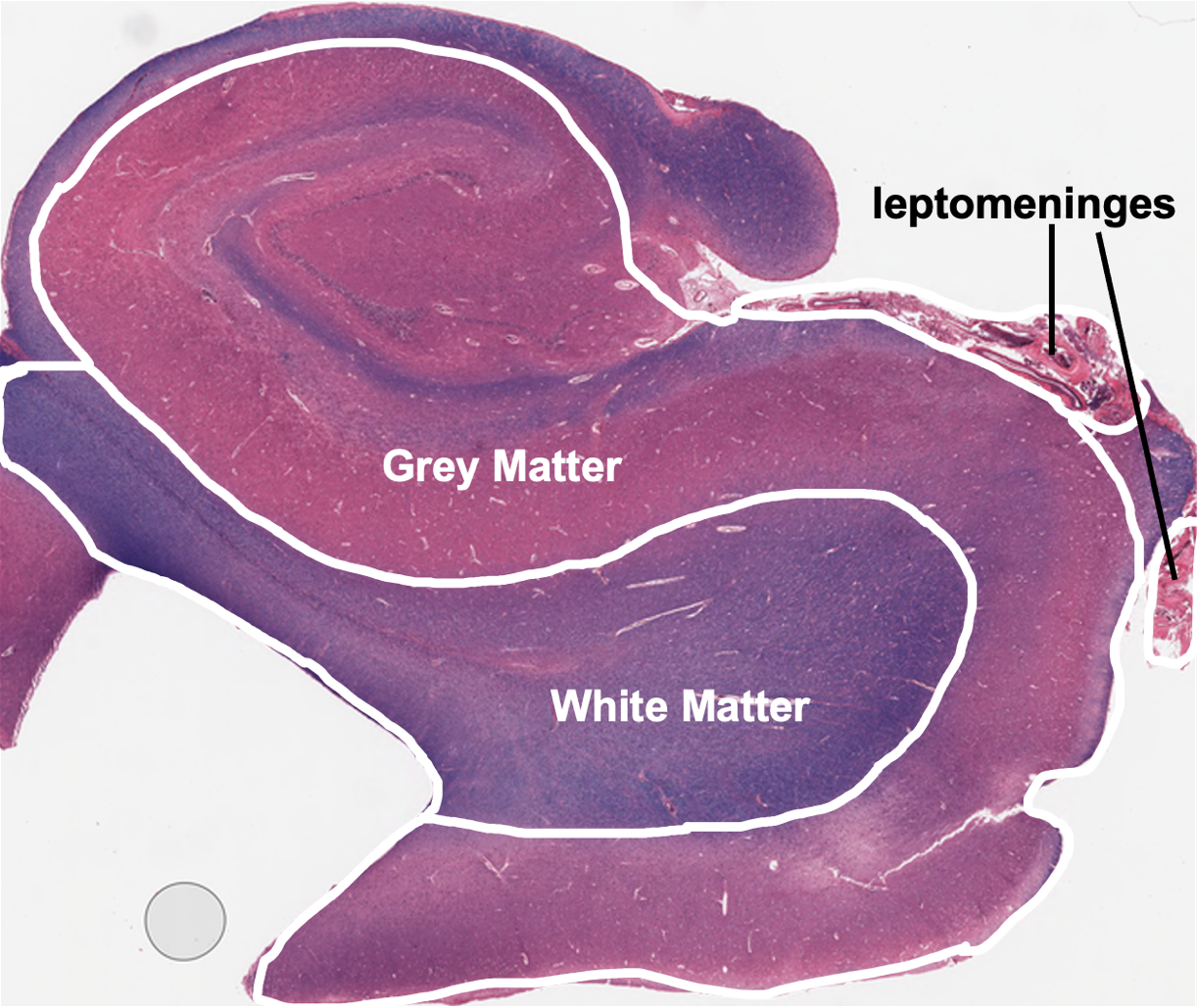


# Supplementary Fig. 4 | Example of segmentation of the gray matter, white matter, and leptomeninges for the analysis shown Figure 3c.
