# Supplementary material for: Histopathologic brain age estimation via multiple instance learning
Source: Acta Neuropathol. 2023 Oct 10;146(6):785–802. doi: 10.1007/s00401-023-02636-3 (PMC10627911; doi:10.1007/s00401-023-02636-3)
Supplement: Supplementary file 2 — Supplementary file2 (DOCX 14 KB) [file 401_2023_2636_MOESM2_ESM.docx]

| **Supplementary Table 1 \| Model Performance as a function of number of attention heads** | | | |
| --- | --- | --- | --- |
| **Number of Attention Heads** | **MAE** | **RMSE** | **MSE** |
| 1 | 5.53 ± 0.30 | 6.90 ± 0.27 | 47.79 ± 5.35 |
| **2** | **5.45 ± 0.22** | **6.80 ± 0.27** | **46.37 ± 3.65** |
| 4 | 5.56 ± 0.28 | 8.22 ± 0.11 | 67.61 ± 1.87 |
| 8 | 5.48 ± 0.28 | 6.83 ± 0.35 | 46.75 ± 5.04 |
| 50 cross-fold runs for the final model and ablation analyses. The final model used in bold  mean and standard deviations are shown.  *MAE*, mean absolute error; *RMSE*, root mean square error; *MSE*, mean squared error | | | |
