# Supplementary material for: Histopathologic brain age estimation via multiple instance learning
Source: Acta Neuropathol. 2023 Oct 10;146(6):785–802. doi: 10.1007/s00401-023-02636-3 (PMC10627911; doi:10.1007/s00401-023-02636-3)
Supplement: Supplementary file 3 — Supplementary file3 (DOCX 14 KB) [file 401_2023_2636_MOESM3_ESM.docx]

| **Supplementary Table 2 \| Number of available cases for the clinical and pathologic variables with HistoAge or DNA methylation data** | | |
| --- | --- | --- |
| **n Cases with Available Data** | **HistoAge** | **DNA Methylation** |
| Total | 689 | 293 |
| Cognitive Impairment Label | 689 | 293 |
| Clinical History of Cognitive Impairment | 538 | 187 |
| MMSE | 200 | 84 |
| CDR | 275 | 168 |
| Cerebrovascular Disease | 227 | 93 |
| Cerebral Amyloid Angiopathy | 513 | 200 |
| ARTAG | 603 | 288 |
| Braak Stage | 689 | 293 |
| Neurofibrillary Tangle Density | 677 | 287 |
| Argyrophilic Grain Disease | 210 | 66 |
| Lewy Bodies | 552 | 246 |
| TDP-43 Positive | 170 | 73 |
| Diffuse Amyloid Plaques | 652 | 278 |
| Neuritic Plaques | 644 | 272 |
